# Platelets in aging and cancer—“double-edged sword”

**DOI:** 10.1007/s10555-020-09926-2

**Published:** 2020-09-01

**Authors:** Alessandra V. S. Faria, Sheila S. Andrade, Maikel P. Peppelenbosch, Carmen V. Ferreira-Halder, Gwenny M. Fuhler

**Affiliations:** 1 Department of Gastroenterology and Hepatology, Erasmus University Medical Center Rotterdam, NL-3000 CA Rotterdam, The Netherlands; 2 Department of Biochemistry and Tissue Biology, University of Campinas, UNICAMP, Campinas, SP 13083-862 Brazil; 3 PlateInnove Biotechnology, Piracicaba, SP 13414-018 Brazil

**Keywords:** Platelet function, Platelet reactivity, Aging, Cancer

## Abstract

Platelets control hemostasis and play a key role in inflammation and immunity. However, platelet function may change during aging, and a role for these versatile cells in many age-related pathological processes is emerging. In addition to a well-known role in cardiovascular disease, platelet activity is now thought to contribute to cancer cell metastasis and tumor-associated venous thromboembolism (VTE) development. Worldwide, the great majority of all patients with cardiovascular disease and some with cancer receive anti-platelet therapy to reduce the risk of thrombosis. However, not only do thrombotic diseases remain a leading cause of morbidity and mortality, cancer, especially metastasis, is still the second cause of death worldwide. Understanding how platelets change during aging and how they may contribute to aging-related diseases such as cancer may contribute to steps taken along the road towards a “healthy aging” strategy. Here, we review the changes that occur in platelets during aging, and investigate how these versatile blood components contribute to cancer progression.

## Introduction

Physiological changes occur in all organ systems during aging, and are a reflection of changes that occur on a molecular level in individual cells. Diverse animal and yeast models have shown that aging is associated with tissue-specific changes in transcriptomes as well as intra- and extracellular metabolite changes [1]. Cellular senescence, a block in cellular proliferation as a result of (amongst others) telomere shortening and loss of DNA damage repair, plays an important role in the process of aging [2]. In addition to telomere attrition, genomic instability, and cellular senescence, other hallmarks of cellular aging include stem cell exhaustion, epigenetic alterations, loss of proteostasis, deregulated nutrient sensing, mitochondrial dysfunction, and altered intercellular communication [3]. Not all cells become senescent, and removal of senescent cells may reduce aging on an organismal level [4]. However, cellular communication is mediated in part via the release of vesicles known as exosomes, which can carry cellular components from one cell to another across large distances. Senescent cells also release such exosomes and these have been speculated to play a significant role in age-related phenotypes including age-related diseases [5]. Connecting all known cellular alterations to biological aging remains challenging, and finding ways to promote “healthy aging” remains a holy grail [3].

Thus far, aging is often studied in the context of stem cell capacity and longevity, but cellular changes in individual cell types have also been investigated for neurons, skin fibroblasts and keratinocytes, bone and bone marrow (bone-proximal osteoblastic niche), and many other tissues and cell types [6–8]. One more cellular component to be added to this mix are platelets, as a role for these blood constituents in aging and age-related diseases is now emerging [9]. Like many systems in cellular metabolism and catabolism, the biology/function of platelets appears to be altered in the elderly. In addition, altered platelet function and clinical conditions such as cancer create a complex chain of cause and effect, which can culminate in systemic responses responsible for the main causes of death in the world, namely, (1) inappropriate blood clot formation known as thrombosis and (2) cancer metastasis, responsible for more than 90% of cancer-related deaths [9, 10]. Thrombotic risk in the elderly is associated with genetic factors, but also with lifestyle, obesity, and diseases such as cancer [11, 12], creating a complex feedback loop. Other examples of the interrelationship between platelet function and pathological conditions can be seen in the acquisition of bleeding disorders such as hemophilia or Von Willebrand syndrome [13], or the involvement of platelets to neurological disorders such as Alzheimer disease (for review, see [14]). In this latter condition, the microenvironment sensitizes platelets to activation and renders them less sensitive to inhibition, most likely due to increased sensitivity to some platelet activation agonists, such as thrombin and collagen, leading to an increase in β-amyloid production by platelets [15, 16]. Large-scale omics studies have demonstrated age-specific proteomic changes in platelets from childhood to adulthood [17], and miRNA patterns associated with age in individuals ranging from 18 to 46 years old [18]. It is conceivable that such cellular changes may predispose an individual to aging-related diseases. In this review, we summarize the impact of aging on platelet function, and investigate how such altered platelet functionality can contribute to aging-related diseases, with particular emphasis on cancer.

## Aging-associated changes in platelet phenotype and function(s)

Since the lifespan of platelets is around 7 to 10 days in the bloodstream, changes in platelet functions may be correlated with megakaryocyte maturation, adhesion, and thrombopoiesis, as changes in megakaryocyte maturation during aging lead to altered proplatelet formation and release of platelets with an altered content [19]. Some of these events appear to be driven by β-adrenergic signals coming from a senescent microenvironment [19–21]. As such, megakaryocyte aging, aging of platelets in the circulation, and cues from an aged microenvironment to megakaryocytes and nascent platelets during organismal aging can all contribute to changes in platelet biology in elderly individuals. Under normal conditions, there is a gradual loss of RNA content over the course of a platelet lifespan, while in aged organisms, distribution of megakaryocyte content to platelets is altered. However, there are also clear differences between “aged platelets” and “platelets in aged individuals.” Hepatic clearance of senescent platelets from the circulation of adult organisms is dependent on the loss of sialic acid residues of glycoproteins in the cell membrane. Activation of the pro-apoptotic BAX–BAK pathway in aged platelets results in caspase-dependent surface exposure of phosphatidylserine, which serves as a recognition signal for phagocytic cells. In terms of functionality, senescent platelets have impaired adhesion and aggregation responses. On the other hand, platelets in senescent organism might be primed to increase their responsiveness to agonists (hyper-reactive platelets) [22, 23].

**Fig. 1:**
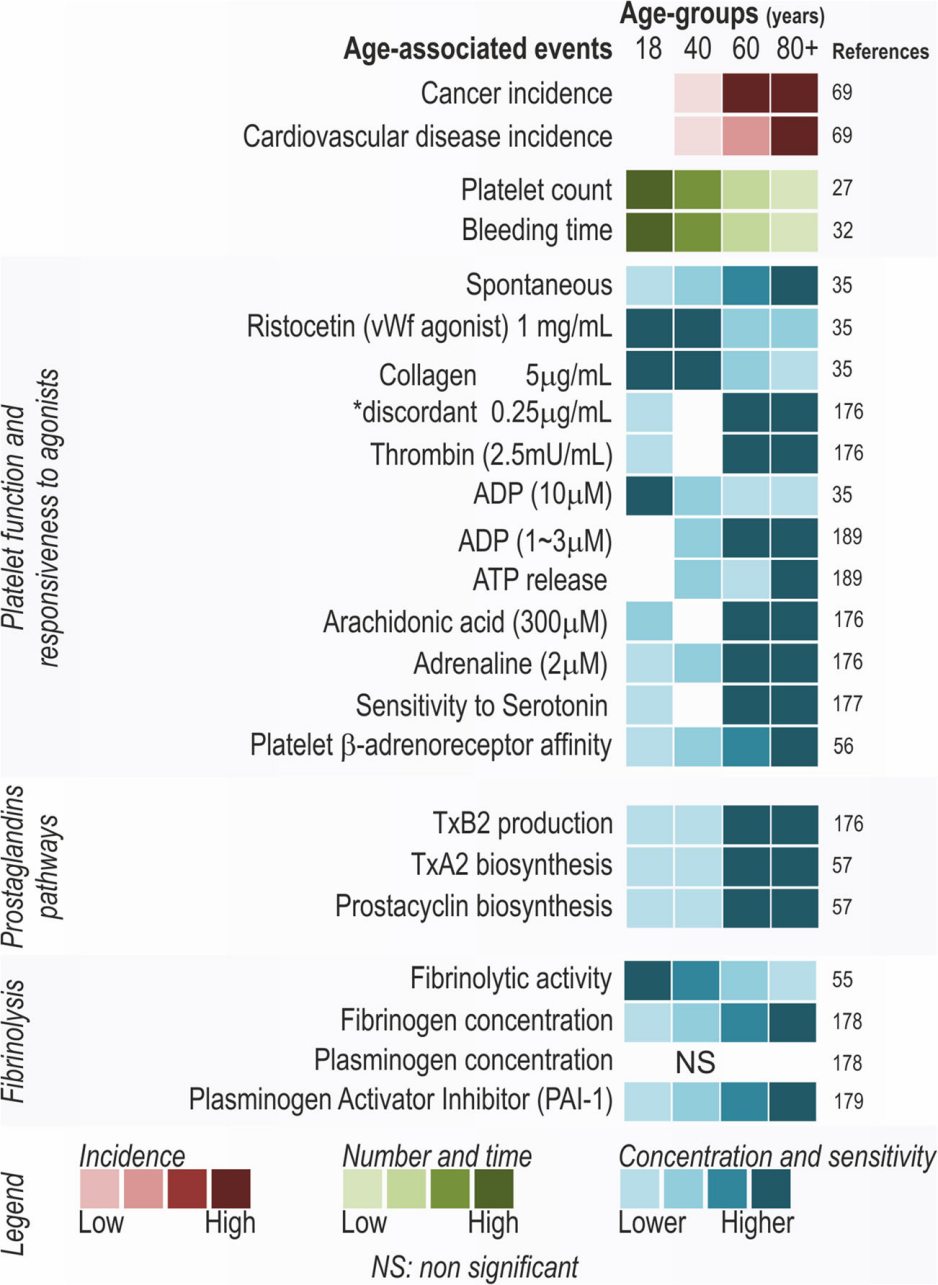
Age-associated changes in platelet function. Platelet function of aggregation, tissue repair, and remodeling changes discriminated on age groups. The concept of age groups is based on young adults (18–39 years), middle-aged (40–59 years), old-aged (60–79 years), very-old-aged group (> 80 years) [27, 29]

**Fig. 2:**
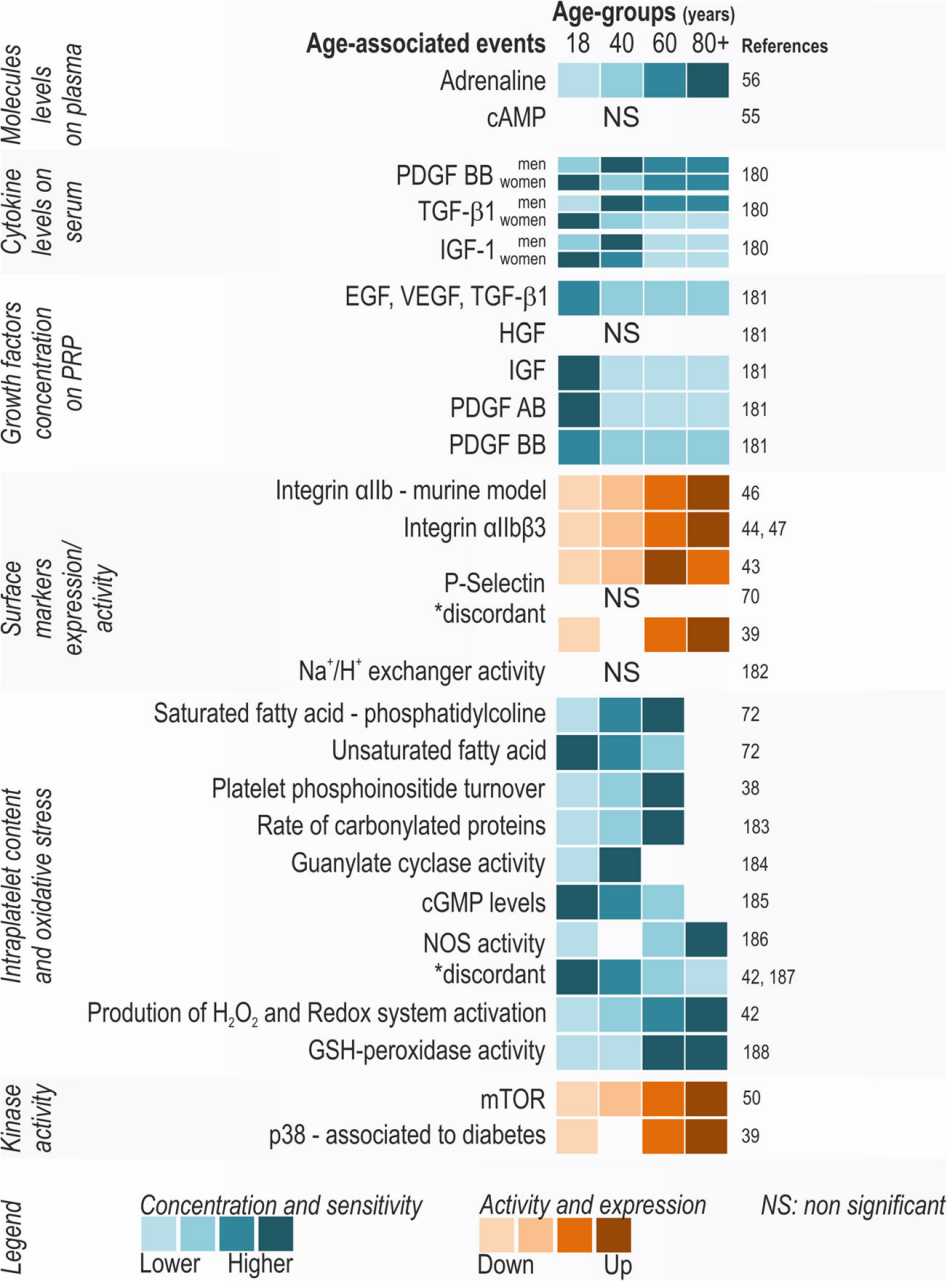
Age-associated changed in platelet markers. Platelets present several changes during the aging process on their content (cytosolic and membrane) and release thereof. The concept of age groups is based on young adults (18–39 years), middle-aged (40–59 years), old-aged (60–79 years), very-old-aged group (>80 years) [40–47, 31, 32, 48, 58]

Several recent studies have investigated the effect of aging on platelet morphology and function. During the course of life, platelet size increases [24], which directly affects platelet content, including granules and pro-coagulation factors. Other morphological changes seen in platelets from older individuals include an irregular, less smooth plasma membrane with more frequent ruptures, and an increase of slender pseudopodia [25]. The number of circulating platelets is thought to decrease with advanced age. While a study of over 5000 participants suggested that platelet count in individuals of > 65 years is not affected by subsequent age differences [26], two large studies investigating over 25,000 and 40,000 individuals, respectively, showed that platelet numbers drop from early childhood, are relatively stable in adulthood, and drop again over the age of 60 years old, irrespective of gender and ethnicity [27, 28]. Careful consideration of the age groups studied is essential, and for the purpose of this review, we therefore aimed to compare young adults (18–39 years), middle-aged (40–59 years), old-aged (60–79 years), and very-old-aged (> 80 years) groups, where possible (Figs. 1 and 2). While the cause of reduced platelet numbers during aging remains to be clarified, some studies have suggested changes in hematopoietic stem cells as a pivotal cause of lower platelet counts in advanced age [59–61].

Despite a lower platelet count in older individuals, bleeding times are reduced during aging, which is thought to contribute to an increased risk of blood clot formation [62]. Bleeding time (i.e., time before efficient blood clotting occurs) is dependent on platelet count and vessel contractibility, as well as platelet function, and platelets in the elderly are indeed hyper-reactivated, especially in subjects with associated comorbidities (for review, see [61, 63]). For instance, spontaneous platelet aggregation is higher in very old subjects as compared with old adults [30, 64], and a higher sensitivity to ADP stimulation [10, 65, 66] and thrombin receptor–activating protein (TRAP6) [67] is seen. Several other platelet agonists, including ristocetin, thrombin, and collagen, have received attention but whether responsiveness of platelets towards these agonists is increased or decreased during aging remains disputed (Fig. 1).

Whether overactivation of platelets is a failed compensation mechanisms to make up for the loss of platelet count remains speculative. The mechanisms contributing to higher platelet activity in elderly individuals are still under investigation. It has been suggested that age-related inflammatory and metabolic changes contribute to an increased platelet function in the elderly [66]. Mouse models have shown an increase of hydrogen peroxide concentration in blood, which directly increases platelet activity during aging [67]. In humans, oxidative stress markers in platelets increase from young to middle-aged individuals [30, 68]. Hydrogen peroxide accumulation in platelets could be the result of NADPH oxidase and superoxide dismutase activity, which are associated with an increased integrin αIIbβ3 activity in platelets [68]. Indeed, the expression of surface markers such as integrin αIIb and αIIbβ3 is increased during the course of aging [69, 70]. Thus, overall increased oxidative stress is generally seen during the aging process, contributing to the concept that platelet alterations in aging are associated with an increasing inflammatory state. The oxidative burst triggers activation of the signaling molecule mTOR, a key regulator of lifespan and aging [69]. mTOR activation in turn results in an increased platelet production by megakaryocytes [70]. Moreover, mTOR hyper-activation during aging is associated with increased platelet aggregability and aging-related venous thrombosis risk in mice [59]. Thus, mTOR plays a dual role in platelet hyper-aggregability by increasing the activity of platelets, while oxidative stress further increases platelet reactivity, resulting in an enhanced risk of thrombi formation in the elderly (Fig. 2).

Association between activated platelets and monocytes, as would occur during blood clotting, enhances the formation of aggregates. While there is no impact of age on platelet-monocyte aggregation *per se* in healthy adults [71], higher levels of platelet-monocytes aggregates were seen in patients with acute coronary syndrome [72], and platelet hyper-activation may thus be further exacerbated in disease states. Others have shown that the age-related increases of platelet-derived β-2-microglobulin levels in the serum cause monocyte differentiation towards a less regenerative phenotype, providing a further link between platelet changes during aging and the aging process [73].

A clear association between platelet hyper-reactivity and the occurrence of thromboembolic events exists and may contribute to cardiovascular comorbidities in the elderly [74]. In addition to the direct effect of aging on platelet aggregation described above, this phenomenon has also been attributed to the fact that the production of anti-coagulation factors does not follow the increasing pro-coagulation factor production during aging [11]. Gleerup and Winther showed that, in addition to an enhancement of platelet aggregability, aging provokes a decrease of fibrinolytic activity, further reinforcing the association between lower fibrinolytic activity forming stable thrombus formation and accumulation, an imbalance between thrombotic *versus* fibrinolytic events [75]. The same research group described that adrenaline and sub-concentration ADP-induced canonical platelet activation is enhanced in old and very old individuals, as is the synergistic effect of serotonin on adrenaline-/ADP-induced platelet activation. Adrenaline levels were also augmented in the old and very old groups [76, 77]. This might be a compensatory mechanism for the fact that β-adrenoreceptors from older individuals show higher ligand affinity. This receptor reduces platelet aggregation through the production of cAMP, and a reduced signaling capacity through this receptor may thus contribute to an enhanced platelet aggregation in the elderly; however, the levels of cAMP in plasma did not change significantly during aging [76, 77]. Endothelial dysfunction during aging may further increase platelet responsiveness [75]. For instance, it has been speculated that platelet activation and aggregation caused by dysfunctional lung epithelium in virally infected individuals may cause depletion of thrombocytes, and contribute to the thrombocytopenia observed in COVID-19 patients infected with SARS-CoV-2 [76, 77].

In addition to blood clotting, it is increasingly recognized that platelets play an important role in wound healing. While wound healing is not absolutely impaired, delayed closure rates and weaker wound repair are commonly seen in subjects of advanced age [78]. During wound healing, many different cell types, including fibroblasts and immune cells such as macrophages and lymphocytes, cooperate to restore tissue architecture. Activated platelets trapped in the blood clot release mediators to attract these cells and express P-selectin which acts as cell adhesion molecule for passing lymphocytes [79]. Furthermore, the secretion of several growth factors, such VEGF, PDGF, EGF, and TGFβ, may modulate T cells to induce keratinocyte regenerative capacity and enhance proliferation of regenerative cells such as fibroblasts [80, 81]. However, while reduced serum levels of these platelet-derived factors could theoretically contribute to decreased wound healing rates, age-related variations in cytokine levels appear most pronounced in early adulthood, disputing their relevance for wound healing delay in the very old individuals [25, 82].

Data collection on platelet function during aging is complicated by several issues. For one thing, platelet aging may be gender-specific, as studies have indicated that aging-related loss of interaction with the adhesion molecule von Willebrand factor (vWF) is more pronounced in women as compared to men [28, 83]. Thus, hormonal changes may contribute to platelet alterations in older subjects [84]. Levels of steroids such as testosterone and dihydrotestosterone in older individuals are negatively associated with platelet activation markers, and these steroids can directly inhibit collagen-induced aggregation *in vitro* [85]. Secondly, recent data suggest that changes that occur during aging are complicated and were not always found to be continuous during aging. Spontaneous aggregation was increased in elderly individuals compared with younger subjects, while ristocetin or collagen-induced aggregation was decreased (pointing towards platelet exhaustion) [30]. However, these trends did not follow linear relationships with changes most pronounced in the very old (80+ years) [30]. Other platelet activation markers (soluble P-selectin, integrin αIIb, caspase 3, oxidative stress) were shown to increase from young to old individuals, but decrease again in the very old [68]. However, it should be noted that others found no differences in basal membrane-bound P-selectin between individuals < 45 years and > 65 years old [34, 35], while the percentage of platelets expressing P-selectin upon stimulation with TRAP-6 was actually higher in younger individuals [67]. Differences in age groups, methods, and stimuli used vary per study and may account for conflicting results. It should further be noted that the effects observed are sometimes small, and small group sizes may hamper interpretation of results. While many studies point towards disturbances in platelet functionality during aging, the direct consequences on coagulation in healthy aging may not always be clear [85, 86], and may be more pronounced under pathological conditions.

### Platelet bioactive lipids in aging

A detailed study on platelet lipid production and aging was reported in 1986 [49]. This study investigated platelet cholesterol and phospholipids content, and observed a slight increase of cholesterol/phospholipids molar ratio upon aging within a range of 20 to 69 years old [87]. It is important to highlight that platelets are not able to produce their own cholesterol, which must be obtained during their genesis (from megakaryocytes) or derived from plasma. The cholesterol/phospholipid molar ratio is important to maintain platelet membrane fluidity, and, consequently, the platelet capacity to change its shape during activation. In addition, activation of platelets via agonist-receptor activation in many cases requires localization of receptors and downstream signaling molecules in cholesterol-rich lipid rafts [88]. The lipid composition is also affected by aging [89], with increased fatty acids 16:0 phosphatidylcholine and sphingomyelin, and a decrease of linoleic acids 18:2, 20:4, and 20:3 in older subjects [49]. It is important to note that lipid oxidation occurs on platelet LDL, and this phenomenon may have severe consequences for cardiovascular diseases. One study showed that older males at risk for coronary heart disease due to dietary habits (55–73 years old) showed higher platelet aggregation in response to epinephrine as compared with younger individuals (28–54 years old) and males at lower risk for heart disease, indicating that age-related platelet changes associated with phospholipid content may be a risk factor for cardiovascular diseases [90].

Besides the platelet membrane lipid composition, the most important bioactive lipids relevant to platelet function are the signaling lipids derived from the eicosanoid pathway. Briefly, upon stimulation of cells, membrane-anchored arachidonic acids (AA) are released from the membrane phospholipids by phospholipases (phospholipase A2), after which they are enzymatically converted to prostanoids by COX1/2 enzymes. This process results in production of platelet stimulatory thromboxane (TxA_2_, mainly produced via COX1 [91]) or platelet antagonistic prostaglandins (PG), PGI_2_, prostacyclin), PGD_2_, and PGE_2_ (mainly via COX2) [92, 93]. Alternatively, AA can be converted to leukotrienes through lipoxygenases activity. Eicosanoids are important mediators of inflammation, and, indeed, eicosanoid biosynthesis is higher on advanced age [77, 94, 95], which in turn may contribute to enhanced inflammatory state during aging [92, 94, 96]. Platelet interaction with peripheral blood mononuclear cells directly modulates inflammatory responses, potentially through their production of PGE_2_ [79, 80]. In this case, PGE_2_ decreases the effectiveness of myeloid cell differentiation and affects their responses [97].

However, both increased TxA_2_ as well as PGE_2_ and prostacyclin excretion were seen in older humans or rats, which begs the question of how this balance would affect platelet activity [77, 98, 99]. While TxA_2_ is produced by platelets, the major source of prostacyclins is endothelial cells. While some studies showed no differences in prostacyclin secretion by arterial endothelial cells for donors of different ages [97], others demonstrated reduced prostacyclin expression in aorta endothelia from older individuals, suggesting that perhaps the TxA_2_ effect wins out during aging. It is of interest to know that dietary restriction, known to prolong healthy aging, is associated with an enhanced prostacyclin/TxA_2_ ratio in rats [100, 101]. Indeed, increased TxA_2_ excretion appears to be associated not only with age-related diseases such as atherothrombosis but also with metabolic disease [102, 103]. Obesity and decompensated glucose metabolism increase not only platelet activation but also inflammation (for review, see [104]). In this case, the persistent TxA_2_-dependent platelet activation increases systemic inflammation [103, 105]. Inflammation-induced endothelial events may play a major role in aging comorbidities. For instance, glycemia-mediated TxA_2_-receptor activation was associated to disturbed blood-brain barrier integrity in diabetes [106]. Furthermore, TxA_2_ is a P2X_1_ ion channel agonist and both platelets and P2X_1_ are required to maintain vascular integrity in a mouse colitis model [107, 108].

Taken together, a clear change in platelet morphology and function is seen during aging, which may have severe consequences for aging-related physiology. The most relevant changes in platelet biology were highlighted in Figs. 1 and 2.

## Platelets in cancer—“double-edged sword”?

As described above, platelet hyper-reactivity during aging is associated with an increased risk of formation of embolisms. Nevertheless, despite cancer being an age-related disease, thrombocytopenia is a common event in these patients. The risk of bleeding in thrombocytopenic cancer patients is difficult to predict [109], and platelet counts must be carefully monitored. In particular, cancers of the bone marrow (platelet production from megakaryocytes) or spleen (platelet clearance), where hematopoiesis is affected, are prone to lead to loss of platelet counts. For instance, thrombocytopenia in patients with bone dyscrasias is directly related to bleeding events [110]. However, the most common cause of bleeding due to platelet loss in cancer patients arises as a result of myeloablative chemotherapy [111] and cytopenia may therefore be a bystander effect rather than a pathogenic event. In fact, the role of platelets in cancer appears to be ambiguous, as enhanced blood clotting represents a major risk factor in cancer patients.

Patients with cancer (but also those with cardiovascular diseases including diabetes, hyper-cholesterolemia, and hypertension) can develop an increased platelet activity, which may be either age-related or disease-specific. The hyper-aggregability observed in these diseases appears to be related to higher platelet reactivity towards agonists or increased circulation of these agonists (such as thrombin and factor Xa), and is a primary cause of thrombotic events, in particular venous thromboembolism events (VTE) and arterial thrombosis (AT) [112, 113]. These events partially overlap, with shared risk factors, and similar incidence in cancer patients [114, 115].

The first report of a platelet-related disorder in cancer came from Armand Trousseau, who described a higher risk of thrombotic events in cancer patients [116], which has subsequently been termed Trousseau syndrome. As the second cause of death, VTE poses a significant comorbidity in cancer patients, and a common cause of hospitalizations, thereby significantly contributing to cancer-associated health care costs [117]. Several cancers are associated with increased VTE risk, including renal carcinoma [118]; hepatocellular carcinoma [119]; lung cancer [120]; and esophageal and stomach cancer [112]. Moreover, VTE in esophageal or gastric cancer patients has been associated with decreased survival: patient survival without VTE is 18 months compared with 13.9 months with VTE [121]. While the risk of VTE appears to be especially high in patients suffering from stomach and pancreatic cancer, up to 20% of all cancer patients may develop thromboembolisms, including pulmonary and venous events. For AT, the overall incidence of events in patients with cancer is increased 2-fold [115].

Enhanced platelet activation as determined by mean platelet volume (MPV) is seen in cancer patients, and may correlate with tumor stage [122, 123]. Both MPV and increased soluble P-selectin levels correlate with VTE development in cancer patients [124–126]. Age does not predict VTE risk for all cancer types, suggesting that at least for some cancer types, tumor cells themselves increase platelet reactivity and VTE risk [127]. Indeed, higher platelet P-selectin expression was found in mouse models of breast cancer, which in turn was associated to lung metastasis [128]. In addition, MPV, which is enhanced in malignant tumors, drops upon treatment [129], enforcing the direct link between tumor burden and platelet activation. Thus, cancer cell–mediated platelet hyper-reactivity contributes to increased VTE risk. While to date, there is no method available and validated to monitor the clinical implication of platelet hyper-aggregability in cancer patients; this may be a promising avenue of investigation [130].

Multiple mechanisms may underlie the tendency of platelets from cancer patients to aggregate. Tumor cells can stimulate platelet aggregation through direct interaction via adhesion molecules or via the delivery of extracellular vesicles and/or secreted factors. This phenomenon, described as tumor cell–induced platelet activation (TCIPA), was already identified decades ago [130]. It has now been shown that single tumor cells are capable of attracting and activating platelets to form fibrin clots [131]. Furthermore, platelets from cancer patients differ from platelets from healthy controls in their mRNA profiles, with mRNA transcripts undergoing alternative splicing under influence of tumor-derived stimuli [132, 133]. Platelets are also capable of taking up tumor content, as determined by the fact that tumor-specific mutations can be identified in platelets upon co-culture with tumor cells. This process appears to be regulated by extracellular vesicles released by the tumor cells, which are subsequently taken up by co-cultured platelets [134]. This alteration of platelets by tumor cells, i.e., tumor education, was shown to contribute to an increased adhesive propensity of platelets [135–137]. Furthermore, cancer cells shed extracellular vesicles containing the adhesion molecule tissue factor (TF), which may contribute to VTE at sites of vessel damage [134, 138].

## Platelets drive tumor growth, angiogenesis, and metastasis in cancer

Specifically in solid tumors, the interaction of tumor cells and platelets leads to a condition called paraneoplastic thrombocytosis, in which malignant tumors not only hijack or mimic platelet functions but can also increase their production. A cyclic picture emerges, which contributes to the most feared outcome of a malignant neoplasm: metastasis [139]. Metastasis is the principal cause of death in cancer patients and investigation of the molecular mechanisms that coordinate this process is therefore crucial. The process of metastasis requires several steps: invasion of cells in the surrounding matrix, intravasation to the blood circulation, survival at the circulation, extravasation at the secondary site (tissue or organ), micrometastasis formation and colonization [140]. The primary tumor can shed many cells during the growth phase; however, only a few cells are able to colonize a secondary site [135]. Much depends on the survival of these tumor cells in the blood circulation, survival of detachment, and the hemodynamic flux force, as well as escaping the immune system. One of the principal strategies of cancer cells to survive in the circulation is interaction with platelets, and nearly all processes of cancer metastasis appear to be facilitated by interaction of tumor cells with platelets.

Platelets can stimulate expression of metalloproteinases in tumor cells, which in turn contributes to tumor cell invasion by facilitating extracellular matrix degradation [141, 142]. Tumor cell metastasis often requires the acquisition of a different phenotype, termed epithelial-to-mesenchymal transition (EMT). This process is characterized by upregulation of several molecular markers (e.g., expression of SNAIL, vimentin cadherin, and MMPs), and platelet-released TGFβ can significantly enhance the upregulation of these markers in cancer cells [143, 144]. In addition, direct contact between cancer cells and platelets contributes to TGFβ/Smad and NFκB pathway activation, culminating in EMT stimulation. Adherence of cells to the extracellular matrix provides survival signals, which are disrupted upon detachment of cells, thereby leading to anoikis: detachment-induced apoptosis. While cancer cells have several mechanisms to overcome anoikis, it has been demonstrated that interaction of cancer cells with platelets further induces tumor cell resistance against anoikis [129]. Thus, platelet-induced alteration of cancer cell intracellular programs contributes to tumor invasiveness and metastasis [135, 144, 145].

Extravasation of tumor cells from tissue to bloodstream is facilitated by platelet-derived ADP stimulation of P2Y_2_ receptors on endothelial cells [146]. Once the cancer cell enters the blood circulation, the dissemination efficiency also depends on the interaction with platelets, with many studies showing that platelets facilitate the metastatic process via hematogenous dissemination [143, 147]. Survival of tumor cells in the blood stream is not only enhanced by platelets through mechanic protection from shear force but also by protecting the cancer cells from circulating immune cells, which may target neoantigens, expressed by tumor cells. Interestingly, it has been demonstrated that cancer cells may mimic platelets by expressing megakaryocytic genes and expressing platelet surface markers, including adhesion molecules such as integrins and selectins [139, 148]. Additionally, coating of tumor cells with platelets allows transferring their major histocompatibility complex (MHC) class I to tumor cells, thereby giving these cells a false “pseudonormal” exterior, and allowing escape from immunosurveillence by natural killer cells [149]. TGFβ released by platelets also downregulates the NK receptor NKG2D on tumor cells, further shielding them from immunosurveillence [150, 151]. Lastly, extravasation of the tumor cells from the blood stream is facilitated by platelets, and appears to require binding of platelets to Integrin ανβ3 expressed on tumor cells [152].

As a solid tumor grows and its oxygen and nutrient demands increase, angiogenesis, the formation of new blood vessels, is essential for its survival. Tumor-induced angiogenesis often results in an abnormal vasculature with suboptimal perfusion. Nevertheless, tumor cells may benefit from this, as this may reduce delivery of therapies and tumor-targeted immune cells [150]. Furthermore, tumor cells may adapt to such ineffective vascularization, and the ensuing hypoxia may favor tumorigenesis by selecting for aggressive and metastatic clones [153]. Supplementation of platelets or their released products stimulates angiogenesis induced by breast tumor cells *in vitro* [136, 154]. In glioblastoma patients, release of VEGF by platelets was shown to contribute to vessel formation [155], although other studies indicated that platelet-induced angiogenesis was independent of VEGF but most likely relied on release of several other factors, including IL6, thrombopoietin, and angiopoietin [156, 157]. Furthermore, animal models indicate that tumor-educated platelets are more efficient at inducing angiogenesis than healthy platelets, suggesting a more efficient delivery of pro-angiogenic factors by tumor-educated platelets [158]. This appears to be supported by findings in humans, showing that levels of VEGF are increased in platelets from prostate, breast, and colorectal cancer patients [159, 160]. It is of interest to note that vasculogenic mimicry, where tumor cells themselves rather than endothelial cells form vessels, is inhibited by platelets. While counterintuitive, this process is thought to promote metastasis [161]. Thus, platelets tightly coordinate the vascularization process in the context of cancer, and may thereby potentiate malignancies.

Thus far, platelet participation in cancer progression has been associated with vascularization, delivery of growth factors, and hematogenous dissemination [143]. In addition, platelets may directly stimulate cancer cell proliferation through upregulation of oncogenic genes, as was demonstrated for colorectal cancer cells [131]. Thus, platelets play a role in all aspects of cancer progression, something we may do well to take into account when addressing these diseases.

Taking the above into account, it is perhaps surprising to realize that fibrinolysis, the process of dissolving a blood clot, can also play a tumor-promoting role [162]. The main enzyme promoting fibrinolysis is plasmin, while the platelet-derived plasminogen activator inhibitor (PAI) is the main suppressor of this system. Elevated PAI-1 levels are associated with VTE [163], and may explain VTE in pancreatic and glioma cancer patients [164, 165]. As such, inhibition of fibrinolysis is detrimental to cancer patients. On the other hand, plasminogen itself contributes to metastasis by degradation of the extracellular matrix surrounding tumor cells. In addition, the fibrinolytic system contributes to inflammation, angiogenesis, the release of tumor growth factors, and other tumor-promoting functions [162]. Thus, coagulation and fibrinolysis play double roles in cancer, highlighting platelet performance as double-edged sword [166].

**Fig. 3:**
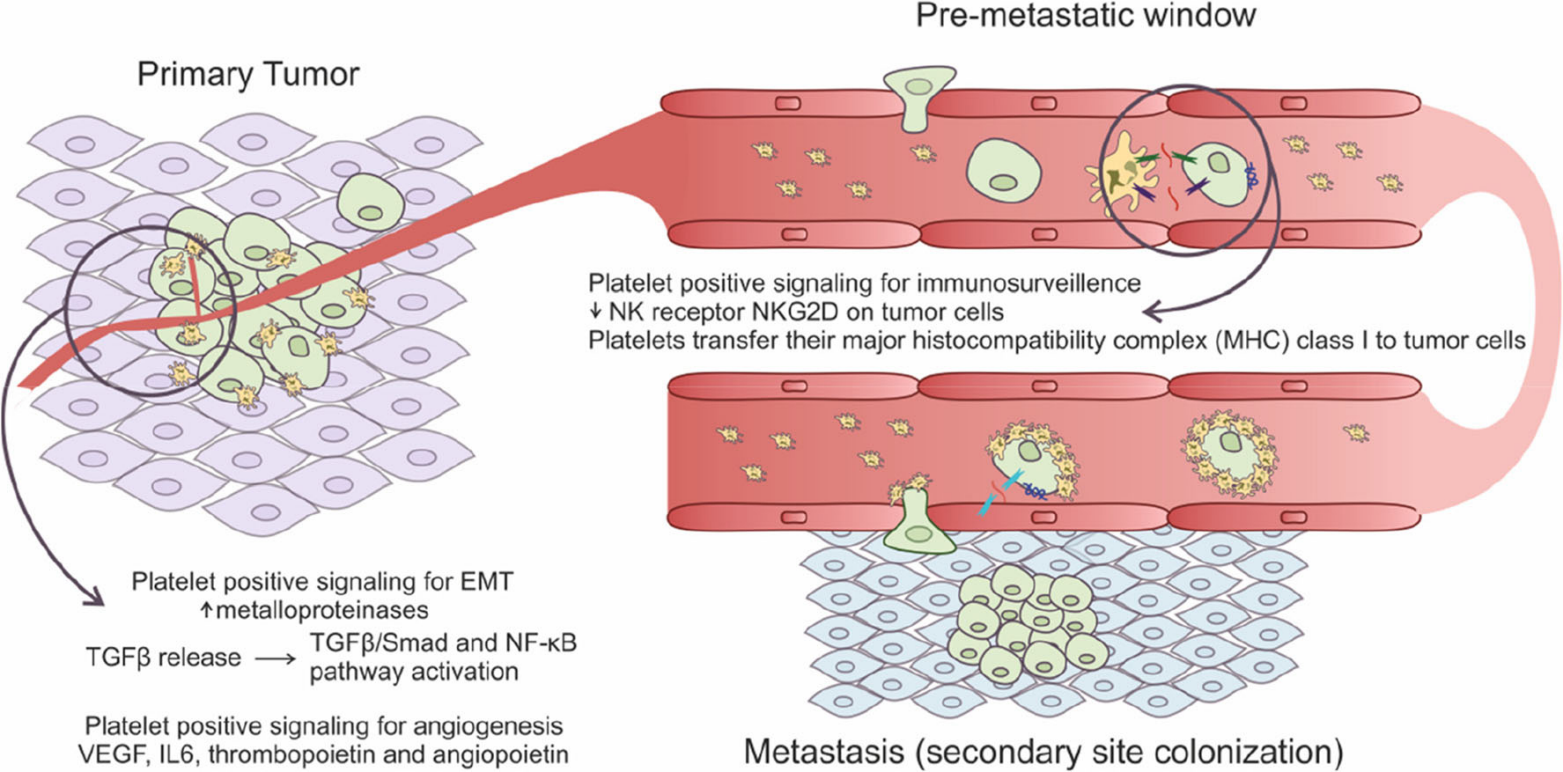
The cross talk between cancer cells and platelets support metastasis, angiogenesis, and tumor growth. Platelets release factors such as TGFβ and VEGF that stimulate epithelial-to-mesenchymal transition (EMT) and angiogenesis. Additionally, platelets contribute to escape from immunosurveillance by covering cancer cells and shielding them from the immune system

In order to target these interactions in healthy aging as well as age-related diseases, detailed knowledge regarding the molecular mechanisms involved may prove essential (Fig. 3). Many of the molecular interactions between cancer cells and platelets depend on their molecular cell surface composition. Platelets can interact with cancer cells via tissue factor (TF), selectins, integrins, and glycoproteins receptors, all of which may activate signaling pathways leading to platelet activation. Thus, platelet membrane components have multiple functions: they contribute directly to hemostasis during thrombus formation, but can also contribute to multifactorial cancer dissemination. TF expressed by cancer cells stimulates platelet activation and initiation of the coagulation cascade. The fibrin produced by platelets subsequently interacts with integrins from cancer cells as well as platelets themselves, inducing formation of cancer cell–fibrin–platelet clusters, which may enter the circulation [167, 168]. Overexpression of TF on breast cancer cells has been reported, and appears to be linked to the release of TGFβ from activated platelets [169]. Furthermore, in ovarian cancer, platelet-induced increase in TF acts as a metastasis initiator [170].

The contribution of integrins to cancer cell–platelet interactions is broad and bidirectional. Platelets express integrins αIIbβ3, αvβ3, α2β1, α5β1, and α6β1, which bind preferentially fibrinogen, vitronectin, collagen, fibronectin, and laminins, respectively, all of which have been described to have adhesive proprieties [150]. Mammadova-Bach and colleagues described that integrin α6β1 from platelets directly binds ADAM9 from tumor cells, a member of the disintegrin and metalloproteinase family. As a consequence of this interaction, platelets are activated and support hematogenous dissemination of cancer cells [171]. Conversely, as already mentioned above, interaction of αvβ3 on platelets was associated with extravasation in aggressive breast cancer [152]. A last class of molecules facilitating the interaction between cancer cells and platelets are selectins, membrane-localized glycoproteins that bind carbohydrates from glycoproteins, glycolipids, and glycosaminoglycan/proteoglycans. Of the selectin family, P-selectin is expressed on platelets and endothelial cells and has already been mentioned above. Platelet dysfunction as a result of P-selectin deficiency limits colon carcinoma and metastasis progression [172, 173]. E-selectin, which is produced by endothelial cells, binds to sialyl-Lewis-x/an, otherwise known as CA19-9, a common tumor marker. The ensuing interaction promotes hematogenous dissemination of colorectal cancer cells [174].

Platelet bioactive lipids are also associated to cancer metastasis (for review, see [175]), and prostanoid synthesis inhibition as a strategy for cancer treatment has been suggested since 1972 [176]. Leukemic cell–induced platelet aggregation is associated with increased TxA_2_ and decreased leukotriene B4 (LTB-4) production by platelets [177]. TxA_2_ in turn promotes metastasis of various tumor models by increasing TCIPA, endothelial cell activation, and recruitment of innate immune cells, all contributing to creating a pre-metastatic niche [178]. Targeting COX1/TxA_2_ appears efficient to reduce tumor cell metastasis [179, 180]. Conversely, prostacyclin, one of the most potent platelet inhibitors, prevents metastasis in a melanoma model [176, 178]. Endothelial function, essential to tumor cell intravasation/extravasation, is also modulated by prostacyclins. Interestingly, endothelial dysfunction, as characterized (amongst others) by decreased prostacyclin and increased P-selectin levels, was associated with more severe lung cancer stage, but also to patient age [181]. PGD_2_ can also decrease tumor MMP-2 expression, inhibit EMT inhibition, and reduce tumor cell proliferation [182, 183]. While these latter functions appear to be independent of platelets, some of the prostacyclin-mediated anti-tumor effects may come from inactivation of platelet hyper-reactivity in response to cancer cells, as was shown for melanoma, lung cancer, and breast cancer [179]. However, the anti-tumorigenic effects of prostacyclin and PGD_2_ may be specific to these prostanoids, as PGE_2_ did not reduce TCIPA, and COX2 and PGE_2_ have been associated with enhanced rather than reduced cancer metastasis [184, 185]. Thus, while COX2 inhibitors have been advocated as anti-cancer treatments in the context of inflammation (i.e., prostaglandins are important mediators of inflammation, which in turn may have carcinogenic effects), caution should be taken [186, 187]. Complicating matters further is the fact that platelets and their products may actually protect endothelial cells, in particular under inflamed conditions (e.g., platelet dysfunction has been suggested to contribute to endothelial dysfunction in COVID-19 patients) [188]. By strengthening the endothelial barrier, platelets may prevent intra/extravasation of tumor cells, thereby limiting tumor metastasis (reviewed in [189]).

All in all, many different molecular associations underlie platelet–cancer cell interactions and a better insight into these pathways may provide targets for treatment of both cancer and its associated VTE risk in elderly patients. With platelets playing multiple roles in cancer progression, care needs to be taken when using platelet inhibitors [189].

## Conclusions

**Fig. 4:**
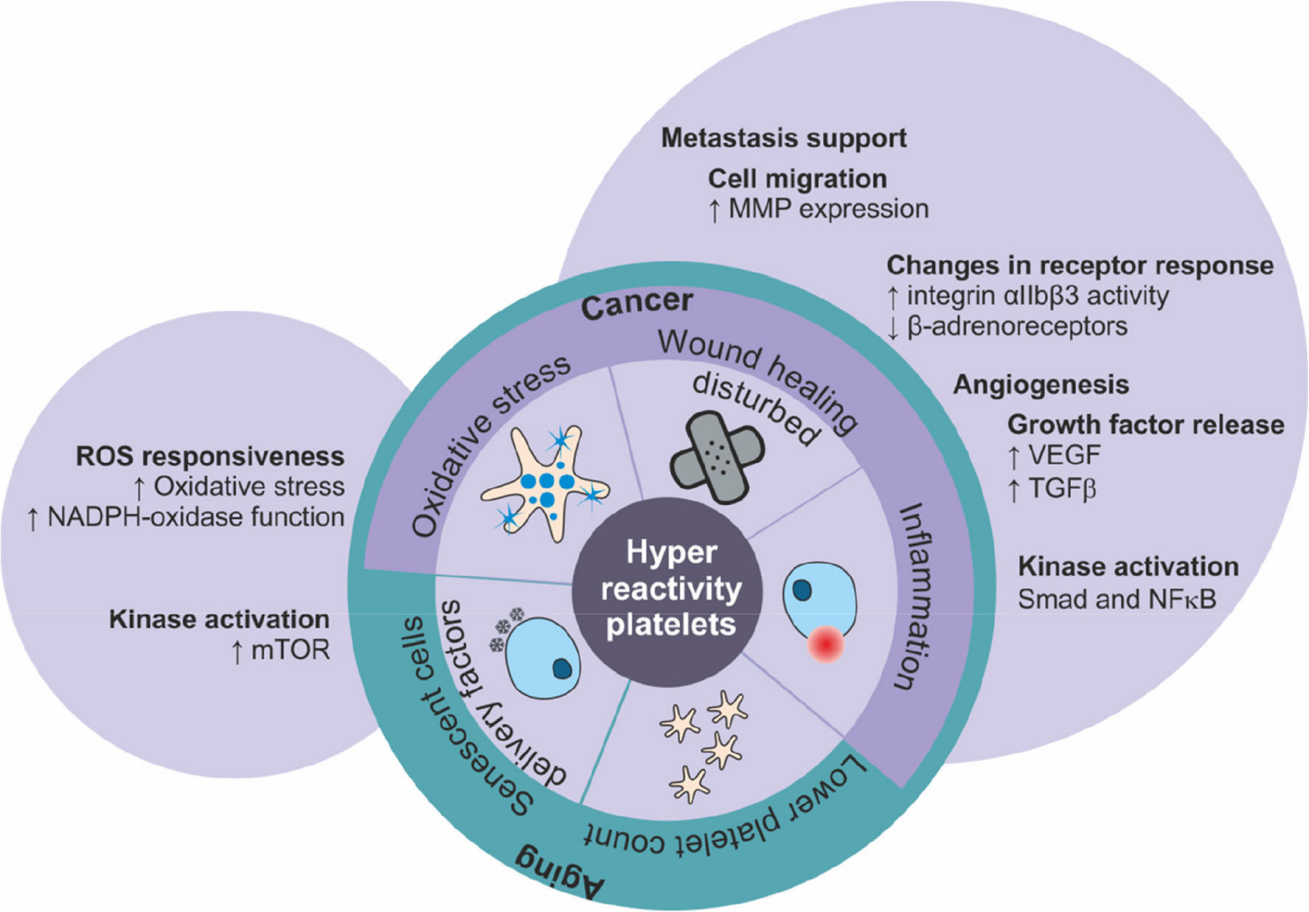
Aging-related changes in platelet function and their association with aging-related diseases (e.g., cancer). As a cross-link between aging and cancer, oxidative stress, wound healing disturbed, inflammation, lower platelet count, and senescent cells delivery factors are highlighted. Platelets support metastasis by augmentation of integrin activity, increasing expression of metalloproteinases, and the release of growth factors, which also augment angiogenesis. Furthermore, kinase activation, including mTOR pathways, increase platelet activation. Production of reactive oxygen species enhances platelet production

It is becoming increasingly clear that aging is associated with changes in platelet ontogenesis/biogenesis and function, and that this may have consequences for physiological aging. With the (relatively late) recognition of the importance of platelets, it has also become evident that age-related diseases such as cancer and cardiovascular disease are associated with platelet alterations (Fig. 4). However, to what extent this is driven by age-related changes or whether these alterations are disease-specific is perhaps unclear and age-matching in platelet investigation is imperative. Nevertheless, evidence showing that tumor cells directly modulate platelet content and functions suggests that while aging may predispose towards platelet dysfunction, specific disease states may further exacerbate platelet dysfunction to a pathological extent. Finding ways to break this pathological interaction while maintaining the balance of hemostasis may prove an important step towards healthy aging.
